# HomeCoRe for telerehabilitation in mild or major neurocognitive disorders: a non-inferiority randomized controlled trial

**DOI:** 10.1007/s11357-025-02006-9

**Published:** 2025-11-13

**Authors:** Sara Bernini, Alice Valcarenghi, Silvia Panzarasa, Silvana Quaglini, Alfredo Costa, Matteo Cotta Ramusino, Marta Picascia, Marica Barbieri, Cristina Tassorelli, Tomaso Vecchi, Sara Bottiroli

**Affiliations:** 1 IRCCS Mondino Foundation, Via Mondino 2, 27100 Pavia, Italy; 2 Department of Psychology, University “G. d’Annunzio” of Chieti-Pescara, Via Dei Vestini 31, 66100 Chieti, Italy; 3 Department of Electrical, Computer and Biomedical Engineering, University of Pavia, Via Adolfo Ferrata 5, 27100 Pavia, Italy; 4 Department of Brain and Behavioral Sciences, University of Pavia, Viale Golgi 19, 27100 Pavia, Italy; 5 Dementia Research Center, IRCCS Mondino Foundation, Via Mondino 2, 27100 Pavia, Italy; 6 Headache Science & Neurorehabilitation Center, IRCCS Mondino Foundation, Via Mondino 2, 27100 Pavia, Italy; 7 Applied Psychology Research Unit, IRCCS Mondino Foundation, Via Mondino 2, 27100 Pavia, Italy

**Keywords:** Cognitive decline, Mild neurocognitive disorder, Dementia, Computerized cognitive training, Non-pharmacological intervention

## Abstract

This randomized controlled trial (RCT) assessed the non-inferiority of HomeCoRe, a home-based version of the CoRe (Cognitive Rehabilitation) software, compared to its in-clinic counterpart in older adults with mild or major neurocognitive disorders. Seventy-seven participants were randomized to receive a 6-week cognitive training with either HomeCoRe (*n* = 40) or CoRe (*n* = 37). Global cognition, specific cognitive domains, and well-being were assessed at baseline and after training. Participant-centered outcomes were also examined, including Weighted Scores, which offer a composite measure capturing both individual session performance and overall rehabilitation progress. Results showed that HomeCoRe was non-inferior to CoRe in improving cognition. Both groups showed significant gains in working memory (*p*_HomeCoRe_ = 0.03; *p*_CoRe_ = 0.005) and logical-executive functions (*p*_HomeCoRe_ = 0.003; *p*_CoRe_ = 0.002), while only HomeCoRe was found to improve global cognition (*p* = 0.01). Weighted Scores significantly increased in both groups (*p*_HomeCoRe_ = 0.001; *p*_CoRe_ < 0.001). No significant differences emerged in mood or mental health outcomes. This study provides important evidence on how the mode of delivery can influence cognitive rehabilitation outcomes, representing one of the first RCTs to assess the same intervention delivered both in-person and remotely. The demonstration of HomeCoRe’s non-inferiority highlights the potential of this promising alternative to in-clinic rehabilitation, promoting accessibility, engagement, and autonomy. Clinicaltrials.gov https://clinicaltrials.gov/ct2/show/NCT04889560 (registration date: May 17, 2021).

## Background

Evidence from the literature suggests the effectiveness of cognitive training programs in people with initial cognitive decline [1–3], i.e., mild (mNCD) and major (MNCD) neurocognitive disorders [4]. Recent advances in technologies have prompted the delivery of treatment services remotely, defined as telerehabilitation (TR) [5–7].

Despite the multiple advantages of TR, several issues are slowing its integration into clinical practice. A major problem is the limited technological proficiency among older adults, often worsened by sensory disabilities such as vision or hearing impairment [8, 9]. This population also tends to exhibit greater distrust and a more negative attitude towards new technologies than younger individuals, which could interfere with the acceptance and autonomous management of TR tools [8–10]. Data on the usability and feasibility of TR devices remain limited. Crucially, usability should be a priority in the development of a TR system [11, 12], as effectiveness should only be assessed once a tool is confirmed user-friendly, intuitive, and engaging, ensuring that future users find the system both accessible and enjoyable [12].

Different TR tools include online platforms [13–15] or installable software applications for computers or tablets [16, 17]. Previous studies considered as an inclusion criterion owning a digital device [16, 17], having full access to an internet connection [13], or both [14, 15], which may have limited the inclusion of less digitalized individuals, excluding a part of the interested population.

Notably, caregivers play an important role in the sustainability of remote healthcare [18], supporting patients during TR sessions and with the training schedule [17]. In the absence of caregivers, a previous study [13] engaged research assistants to monitor and offer support to participants remotely through video calls. However, this solution imposes constraints on both the therapist and the patient, as it requires the simultaneous presence of both agents during the rehabilitation sessions. Consequently, the absence of caregivers may penalize more compromised and less independent individuals, who would not be eligible for TR services. Besides, caregiving has been associated with physical and psychological strain [19]; therefore, attention should be paid to avoid overburdening caregivers to facilitate a sustainable remote healthcare system [18].

To the best of our knowledge, there are a few randomized controlled trials (RCTs) on cognitive TR, but they did not compare the same cognitive intervention delivered through different modalities (e.g., home-based versus in-person). Instead, they investigated the superiority of a cognitive intervention over another, with both experimental and control groups undergoing remote intervention [13–16], without focusing on the delivery modality per se (i.e., TR). Hence, more RCTs aimed at comparing the effectiveness of the same cognitive intervention delivered in different modalities are needed, which would be indicative of the modality-specific effects of delivery.

Another issue is that the evaluation of cognitive TR is heterogeneous [7, 20], with most studies [14–17] focusing on cognitive outcomes, while others prioritize improvements in daily functioning or well-being [13]. This methodological variability affects the comparability of RCTs’ findings, limiting the possibility of drawing definite conclusions regarding the effectiveness of cognitive TR.

Over the past years, we have implemented the software CoRe (Cognitive Rehabilitation) for in-person cognitive training in a clinical setting supervised by a therapist [21, 22]. The system has been successfully evaluated in terms of usability and immediate and long-term effectiveness in people with mild cognitive decline [23–26]. In light of the willingness of treated participants and caregivers to continue the CoRe program at a distance [27, 28], we undertook the development of the home-based version of CoRe (i.e., HomeCoRe), which allows cognitive intervention remotely [29, 30], after a therapist-assisted familiarization session with the tool. We conducted both a preference study comparing the two systems [31] and an evaluation of HomeCoRe’s usability [32], which showed good compliance despite technological familiarity, in line with our previous experience with CoRe [23–25]. Building on this progressive line of research, the present study represents the next step in the development and validation of the CoRe/HomeCoRe systems.

To explore the impact of different delivery modalities for the same cognitive intervention, in this RCT, we evaluated the non-inferiority of HomeCoRe compared to CoRe in older adults with mNCD and MNCD. We hypothesize that cognitive TR provides benefits that are non-inferior to the in-person version of the program. This outcome would support the use of HomeCoRe in the clinical setting, allowing patients to choose the delivery modality that best aligns with their individual needs and preferences.

## Methods

### Participants

Participants were enrolled from the Dementia Research Center outpatient services and Neurorehabilitation Unit of the IRCCS Mondino Foundation (Pavia, Italy), after being screened for eligibility criteria through a clinical evaluation made by an expert neurologist (June 2021–April 2023).

The inclusion criteria were:

- Diagnosis of mNCD or MNCD based on the DSM-5 [33]
- Age between 60 and 85 years
- Years of education ≥ 5
- Clinical Dementia Rating (CDR) [34] score = 0.5–1
- Availability of a caregiver

The exclusion criteria were:

- Mini-Mental State Examination (MMSE) [35] score < 20
- Presence of cognitive impairment secondary to an acute or general medical disorder (e.g., brain trauma or tumor)
- Presence of severe sensory disorder (e.g., deafness or blindness) or motor impairment that prevents trunk control and/or sitting position
- Current cognitive treatments.

### Measures

#### Neuropsychological assessment

The baseline assessment (T0) was performed by using neuropsychological tests standardized for the Italian population, and evaluated the following cognitive domains:

- Global cognition: Mini-Mental State Examination (MMSE) [35], Montreal Cognitive Assessment (MoCA) [36]
- Episodic long-term memory: Logical Memory Test for immediate and delayed recall [37, 38], Rey’s 15 words test for immediate and delayed recall [39], Rey Complex Figure delayed recall [40]
- Logical-executive functions: Raven’s Matrices 1947 [39], Frontal Assessment Battery (FAB) [41], Semantic fluency [37], Phonological fluency (FAS) [39], Rey Complex Figure copy [40]
- Working memory: Verbal Span [38], Digit Span [38], Corsi block-tapping test span [38]
- Attention/processing speed: Attentive Matrices [38], Trail Making Test A and B [42]

The same neuropsychological battery was administered post-training (T1) using parallel versions, when possible, to avoid the practice effect. All the raw scores were adjusted for age, sex, and education, and compared with the values available for the Italian population; then the adjusted scores were transformed into equal scores [43].

#### Questionnaires and scales

At T0, we evaluated functional status (activities of daily living—ADL and instrumental activities of daily living—IADL) [44] and cognitive reserve (Cognitive Reserve Index questionnaire—CRIq) [45].

At both T0 and T1, we assessed depressive symptoms using the Beck Depression Inventory (BDI) [46] and health status using the 36-item Short Form Health Survey questionnaire (SF-36) [47].

#### Participant-centered outcomes

To enhance the comparison between the two systems, we also took into account participant-centered aspects related to the intervention, evaluating both subjective and objective outcomes.

At T1, we assessed the participants’ subjective evaluation of TR effects using the Patient Global Impression of Change (PGIC) [48], which is a scale that measures the perceived change in cognitive functioning, autonomy in daily activities, and quality of life after rehabilitation. Participants responded following the general stem “Compared to how you were before treatment” using the following scale: (1) no change (or got worse), (2) almost the same, (3) a little better, (4) somewhat better, (5) moderately better, (6) better, and (7) great deal better.

We also included an objective outcome measure, the “Weighted Score” (WS), which is a key advantage of CoRe/HomeCoRe, calculated directly by the system and allowing for assessment of both the overall outcome of a training session and the global trend of rehabilitation. The WSs are calculated considering the answers’ accuracy (also taking into account the number of aids required by the participant), the execution time, and the exercise difficulty, informing the therapist about each performance of participant in a single value. It should be noted that the WS was not intended as a direct measure of therapeutic efficacy; instead, it was conceived as a transversal index to summarize participants’ performance across exercises and sessions. Its purpose is to provide clinicians with an intuitive overview of treatment trends, allowing them to visualize performance trends across exercises and sessions. This information can support the monitoring and personalization of future rehabilitation cycles, ultimately enhancing patient engagement and adherence. Here, we compared the WSs of the first and the last session of the intervention for each participant. More information about the WS is reported in the study protocol [28].

### Study design

This study was a prospective, single-blind, parallel-group, RCT with a non-inferiority design. Its protocol was previously published [28] and the trial was registered in https://clinicaltrials.gov (NCT04889560). In this context, we present data comparing T0 and T1 performances. Follow-up outcomes and analyses will be published later. All participants who met the inclusion criteria were recruited and underwent an in-person assessment visit (T0) that lasted about 90 min per participant and was carried out at the hospital setting by a neuropsychologist, using the tests listed in the Measures section. After T0, participants were randomized to one of two groups: HomeCoRe or CoRe. For the allocation, we generated random numbers from a uniform distribution in the range 0–1, dividing the range into two equal intervals and assigning each participant to the group corresponding to the sampled number (1:1 ratio). For both groups, the intervention consisted of a 6-week program (3 sessions/week, each lasting ∼ 45 min). The duration was chosen in accordance with evidence on cognitive interventions in older adults with cognitive decline [49–51] and based on our prior experience with the CoRe software [23–25]. The CoRe intervention was performed under therapist monitoring at the clinic, while HomeCoRe was performed under caregiver monitoring at home, for which remote technical support was available when requested. Before the beginning of HomeCoRe intervention, participants and their caregivers underwent a familiarization session at the clinic during which they were trained on the use of the rehabilitation tool at home. One post-training assessment visit of 90 min for each participant was foreseen at T1, conducted at the clinic by a neuropsychologist, who was blinded to participant allocation.

### Interventions

The cognitive intervention was delivered via the HomeCoRe and the CoRe systems, and it consisted of individual sessions aimed at stimulating several cognitive domains (e.g., logical-executive functions, attention/processing speed, working memory, and episodic memory). Both CoRe and HomeCoRe are research software tools developed within a long-lasting collaboration between clinicians from the IRCCS Mondino and bioengineers from the University of Pavia. Both tools support a participant-tailored intervention through a series of exercise sessions. After the therapist defines the treatment plan, the exercises are delivered in an adaptive manner throughout all sessions, based on the participant’s performance. More specifically, as tasks are dynamically generated, the past performance of each participant, measured through the WS, is analyzed to set the appropriate difficulty level. Each exercise and each level is defined by preset parameters and thresholds used to allow difficulty levels to automatically increase to stimulate neural plasticity [52–54].

Both programs require a personal computer equipped with a touch screen. HomeCoRe was installed on a laptop (password-protected and encrypted) that was supplied to participants by the therapist, whereas CoRe was installed on a desktop computer located in the clinical setting. The software was installed by an expert engineer and under the supervision of the Information Technology (IT) department—IRCCS Mondino.

Compared to CoRe, HomeCoRe exhibits several notable, distinctive features, given its TR role. Firstly, the treatment plan is remotely set up and monitored for treatment adherence and rehabilitation progress by the therapist. The interface of the participant/caregiver is very simple, and it allows viewing and executing the exercises of the day and sending the results to the therapist. It is also equipped with a “caregiver area” where the caregiver can access and communicate via messages with the therapist. Finally, the HomeCoRe system can be used online or offline in case the participant has no access to the Internet. In the online mode, the data communication between the therapist and the participant is managed by the HomeCoRe server, whereas in the offline mode, it is required to manually upload the therapeutic plan and then save the result report on an external memory support (e.g., USB key or hard disk), while the communication with the therapist is asynchronous.

### Statistical analyses

The primary outcome was the change in MMSE at T1 compared to T0. The MMSE was selected as the primary outcome, as prespecified in the study protocol [28], given its widespread use in studies evaluating technology-based cognitive interventions [5, 20], thus allowing for comparability with previous literature.

The secondary outcomes were changes between T0 and T1 in all five cognitive domains, calculated on mean equivalent scores, questionnaires and scales, including participant-centered outcomes to assess subjective evaluation of intervention effects at T1.

The non-inferiority margin (Δ = 2 MMSE points) was selected based on published estimates of the minimal clinically important difference (MCID) in cognitive rehabilitation studies. Meta-analytic data report MCID values ranging from 1.4 to 2.0 points [55], while longitudinal clinical cohorts indicate clinically relevant changes of approximately 1–3 points [56]. Therefore, a 2-point margin aligns with established standards. Based on this margin, we anticipated a between-group difference of ~ 1 point with a standard deviation of 1 point. Using α = 0.025 (one-sided) and 90% power, the required sample size was calculated as 18 participants per group, in line with previous evidence in the literature [57].

Non-inferiority was assessed by calculating the adjusted mean difference between groups on post-training MMSE using an analysis of covariance (ANCOVA) with baseline value and possible between-group differences at T0 as covariates. Non-inferiority was concluded if the lower bound of the 95% confidence interval (CI) of the group difference was above the pre-specified margin (Δ =  − 2 MMSE points).

Separate ANCOVAs were performed for each cognitive domain to examine the effect of group allocation on post-training assessment, while controlling for both baseline performance and possible between-group differences at T0. Pairwise post-hoc comparisons were performed with Holm-Bonferroni correction, and effect sizes were considered as Cohen’s d (*d*).

Considering the non-normal distribution of the collected measures, we used non-parametric tests as exploratory within-group analyses to identify potential changes within each group. As these analyses were exploratory, no corrections for multiple comparisons were applied; therefore, we accepted a certain risk of false positives. The findings from these analyses will require confirmation in future work. For between-group comparisons, the Mann–Whitney test was used for continuous variables and the Chi-square test for nominal variables. The Wilcoxon rank-sum test was used for inter-group comparisons of participants’ characteristics and to evaluate the outcome temporal variations. An effect size index (rank-biserial correlation, *r*₍*rb*₎) was calculated to assess the magnitude of the treatment effect.

Missing data were handled primarily through available-case analysis. As a sensitivity analysis, we applied a Last Observation Carried Forward (LOCF) approach, imputing baseline values for missing T1 assessments. Attrition was minimal (*n* = 6; 3 per group) and balanced.

Only participants with complete data at both T0 and T1 were included in each outcome analysis.

All statistical analyses were conducted using the Jamovi software [58].

## Results

### Participants characteristics

**Fig. 1:**
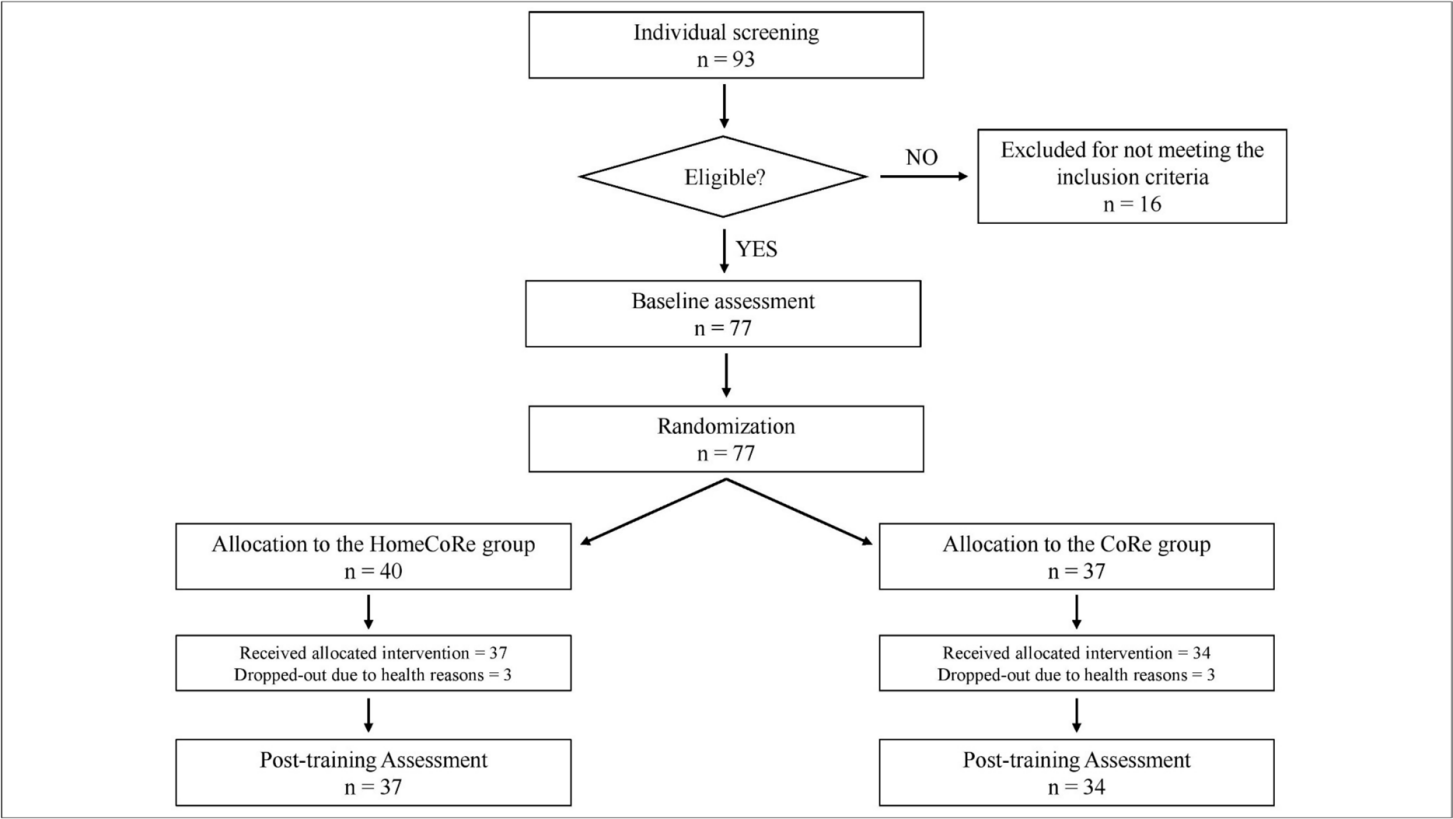
CONSORT flowchart of participants disposition throughout the study

Although the minimum required number of participants was set at 40, enrollment continued beyond this threshold due to the high level of interest in the initiative. A total of 77 participants were enrolled, with 40 randomly assigned to the HomeCoRe group and 37 to the CoRe group. Six patients dropped out between T0 and T1, therefore, analyses were carried out on a final sample of 71 participants: 37 in the HomeCoRe group and 34 in the CoRe group (Consolidated Standards of Reporting Trials–CONSORT–flowchart in Fig. 1). At T0, the two groups did not differ in any demographic or clinical characteristic (Table 1).

**Table 1:** Participant characteristics (means ± standard deviations or percentage of prevalence) at T0 as a function of group

	**HomeCoRe**	**CoRe**	**Mann–Whitney test** ***p* value**
Age	72.46 ± 6.49	73.71 ± 5.69	0.58
Years of education	10.97 ± 4.39	11.06 ± 4.41	0.89
% Female	49%	38%	0.38
% mNCD	86%	97%	0.12
CDRs	0.54 ± 0.22	0.49 ± 0.15	0.22
Cognitive reserve	2.49 ± 1.22	2.32 ± 0.91	0.72
MMSE	26.35 ± 2.48	27.05 ± 2.02	0.32
ADL	5.97 ± 0.16	5.94 ± 0.24	0.52
IADL	7.27 ± 1.41	7.29 ± 0.84	0.27

*Note. T0 =* baseline assessment; *mNCD* = Mild Neurocognitive Disorder; *CDRs* = Clinical Dementia Rating; *CRIq* = Cognitive Reserve Index questionnaire; *MMSE* = Mini-Mental State Examination; *ADL =* Activities of Daily Living; *IADL* = Iinstrumental Activities of Daily Living.

### Baseline evaluation

#### Neuropsychological assessment

As reported in Table 2, the two groups did not significantly differ in MMSE scores (*p* = 0.32) and all the five cognitive domains: global cognition (*p* = 0.33), episodic long-term memory (*p* = 0.09), attention/processing speed (*p* = 0.14), working memory (*p* = 0.47), and logical-executive functioning (*p* = 0.45).

**Table 2:** Neuropsychological assessment at T0 and T1 (means ± standard deviations) as a function of group

	**HomeCoRe**		**CoRe**	
	**T0**	**T1**	**T0**	**T1**
MMSE	26.35 ± 2.48	26.58 ± 2.66	27.05 ± 2.02	27.51 ± 1.91
Global cognition	1.31 ± 0.76*	1.59 ± 0.70*	1.49 ± 0.66	1.69 ± 0.58
Episodic long-term memory	1.17 ± 0.92	1.42 ± 1.10	1.55 ± 0.97	1.79 ± 1.07
Attention/processing speed	2.48 ± 1.10	2.49 ± 1.20	2.07 ± 1.07	2.23 ± 1.04
Working memory	2.59 ± 0.93*	2.95 ± 1.04*	2.48 ± 0.70^†^	2.91 ± 0.71^†^
Logical-executive functioning	2.48 ± 0.84*	2.92 ± 0.71*	2.33 ± 0.79^†^	2.74 ± 0.85^†^

*Note. T0* = baseline assessment; *T1* = post-training assessment; *MMSE* = Mini-Mental State Examination.

^*^Denotes a statistically significant within-group change from T0 to T1 in the HomeCoRe group. ^†^ Denotes a statistically significant within-group change from T0 to T1 in the CoRe group. Results remained unchanged when missing data were imputed using LOCF, supporting the robustness of the findings.

#### Questionnaires and scales

Table 3 indicates that, at T0, the two groups have comparable scores for SF-36 mental status (*p* = 0.13) and BDI (*p* = 0.06), whereas HomeCoRe participants demonstrated better SF-36 physical status scores (*p* < 0.001). Considering this significant between-group difference at T0, baseline SF-36 physical status was considered as a covariate for the between-group analyses on cognitive outcomes at T1.

**Table 3:** Questionnaires and scales at T0 and T1 (means ± standard deviations) as a function of group

	**HomeCoRe**		**CoRe**	
	**T0**	**T1**	**T0**	**T1**
SF-36 physical status	50.13 ± 6.94*	48.06 ± 7.35*	41.27 ± 10.60	43.21 ± 10.57
SF-36 mental status	44.59 ± 9.06	44.63 ± 9.01	41.71 ± 8.68	43.64 ± 11.15
BDI	8.03 ± 5.53	7.68 ± 4.99	11.41 ± 8.05	11.15 ± 6.84

*Note. T0* = baseline assessment; *T1* = post-training assessment; *SF*-*36* = 36-item Short Form Health Survey questionnaire; *BDI =* Beck Depression Inventory.

^*^Denotes a statistically significant within-group change from T0 to T1 in the HomeCoRe group.

### Post-training assessment

#### Neuropsychological assessment

Regarding between-group comparisons, ANCOVA adjusting for baseline MMSE and baseline SF-36 physical status showed no significant difference between groups on post-treatment MMSE (*F*(1, 62) = 0.82, *p* = 0.37, with an adjusted mean difference HomeCoRe—CoRe of − 0.45, 95% CI [− 1.30, 0.40]). As the lower bound of the CI was above the non-inferiority margin of − 2, the pre-specified criterion for non-inferiority was met.

Separate ANCOVAs, adjusting for both baseline scores and baseline SF-36 physical status, revealed no significant between-group differences in post-treatment performance for any of the cognitive domains: global cognition (*F*(1, 62) = 0.0002, *p*_Holm-Bonferroni_ = 0.99, with an adjusted mean difference of 0.002, 95% CI [− 0.55, 0.55]), episodic long-term memory (*F*(1, 62) = 0.001, *p*_Holm-Bonferroni_ = 0.97, with an adjusted mean difference of 0.007, 95% CI [− 0.54, 0.56]), attention/processing speed (*F*(1, 61) = 1.41, *p*_Holm-Bonferroni_ = 0.24, with an adjusted mean difference of − 0.23, 95% CI [− 0.89, 0.23]), working memory (*F*(1, 62) = 0.07, *p*_Holm-Bonferroni_ = 0.79, with an adjusted mean difference of 0.06, 95% CI [− 0.48, 0.62]), logical-executive functioning (*F*(1, 62) = 0.02, *p*_Holm-Bonferroni_ = 0.88, with an adjusted mean difference of − 0.03, 95% CI [− 0.59, 0.51]).

Regarding exploratory within-group analyses, as shown in Table 4, the HomeCoRe group showed no significant changes from T0 in MMSE. A significant improvement in terms of performance at T1 was found in global cognition (*r*₍*rb*₎ = 0.55), working memory (*r*₍*rb*₎ = 0.44), and logical-executive functioning (*r*₍*rb*₎ = 0.58) with respect to T0. No significant differences between T0 and T1 were found in episodic long-term memory and attention/processing speed. The CoRe group showed no significant differences between T0 in MMSE. A significant improvement in terms of performance at T1 was found in working memory (*r*₍*rb*₎ = 0.61) and logical-executive functioning (*r*₍*rb*₎ = 0.65) with respect to T0. No significant differences were reported in global cognition, episodic long-term memory, and attention/processing speed when comparing T0 and T1.

**Table 4:** Neuropsychological Assessment T0 vs. T1 comparisons as a function of group

	**HomeCoRe**		**CoRe**	
	**W**	***p* value**	**W**	***p* value**
MMSE	106	0.21	80	0.13
Global cognition	78	0.01*	104	0.11
Episodic long-term memory	174	0.23	131	0.06
Attention/processing speed	262	0.75	109	0.25
Working memory	149	0.03*	79	0.005^†^
Logical-executive functioning	125	0.003*	87	0.002^†^

*Note. W* represents the test statistics of the Wilcoxon rank-sum test; *T0 =* baseline assessment; *T1* = post-training assessment; *MMSE*  = Mini-Mental State Examination.

^*^ Denotes a statistically significant within-group change from T0 to T1 in the HomeCoRe group. ^†^ Denotes a statistically significant within-group change from T0 to T1 in the CoRe group. Results remained unchanged when missing data were imputed using LOCF, supporting the robustness of the findings.

#### Questionnaires and scales

The HomeCoRe group significantly changed from T0 in SF-36 physical health status (*W* = 346, *p* = 0.02, *r*₍*rb*₎ = 0.49), whereas no difference across testing sessions were found in SF-36 mental health status (*W* = 167, *p* = 0.91) and BDI (*W* = 255, *p* = 0.43). The CoRe group was stable across testing sessions in SF-36 physical health status (*W* = 133, *p* = 0.11), SF-36 mental health status (*W* = 149, *p* = 0.22), and BDI (*W* = 313, *p* = 0.57).

### Participant-centered outcomes

#### Patient Global Impression of Change

The mean total score of PGIC was 3.68 ± 1.33 for the HomeCoRe group and 4.27 ± 1.28 for the CoRe group, without any significant differences between groups (*p* = 0.11), both showing an overall positive perception of improvement in the cognitive status after the intervention.

#### Weighted Scores session 1 vs. session 18

Both the HomeCoRe (*W* = 125, *p* = 0.001, *r*₍*rb*₎ = 0.60) and the CoRe (*W* = 56, *p* < 0.001, *r*₍*rb*₎ = 0.80) groups significantly improved their overall WS performance when comparing session 1 to session 18. The HomeCoRe group reported an average WS of 48.7 ± 3.8 at session 1 and of 54.8 ± 7.8 in the last session of the treatment, and the CoRe group reported an average WS of 49.50 ± 2.9 at session 1 and of 58.30 ± 8.2 in the last session of the treatment.

## Discussion

The present study evaluated the non-inferiority of HomeCoRe compared to CoRe in older adults with mNCD and MNCD, investigating the immediate effects of different delivery modalities for the same cognitive intervention.

The lack of significant between-group differences on the MMSE, together with confidence intervals entirely above the non-inferiority margin, provides evidence that the HomeCoRe system is non-inferior to the CoRe system, supporting its validity as a remote alternative. Moreover, the ANCOVAs performed on all the cognitive domains did not reveal any significant group effects, showing that HomeCoRe achieves comparable cognitive outcomes to its in-person counterpart.

Nevertheless, we conducted additional exploratory analyses to examine potential within-group changes over time, and to investigate whether any specific outcomes showed significant improvements across sessions.

Considering the primary outcome, we found stability in MMSE scores in both groups at T1, which could be attributed to the reduced sensitivity of the test in detecting change [59], particularly in the presence of higher baseline scores. This ceiling effect may limit MMSE’s ability to capture subtle variations in cognitive decline or improvement [60]. Indeed, we found a significant improvement in global cognition—obtained from the mean equivalent scores of MMSE and MoCA—in the HomeCoRe group. This result could be explained by the inclusion of MoCA, which is less susceptible than MMSE to ceiling effects [61] and can detect more subtle cognitive changes [59], thus enhancing the discriminative power of the composite measure of global cognition. Nonetheless, we considered the MMSE as the primary outcome for several reasons. Both HomeCoRe and CoRe are multidomain cognitive interventions; therefore, a measure of global cognition was needed. The MMSE is widely used in literature to assess global cognitive changes [5, 20], which facilitates meaningful comparisons with previous studies and contextualization within the existing body of research, despite our awareness of its limitations in sensitivity and ceiling effects [62]. To complement this measure, we included a composite index of global cognition among the secondary outcomes, thereby increasing sensitivity to subtle cognitive changes.

The significant improvement in global cognition observed in the HomeCoRe group may be attributed to a higher level of engagement in TR compared to the in-clinic intervention. Indeed, TR systems have been shown to enhance participants’ engagement by fostering a sense of independence and empowerment [63]. By enabling individuals to take an active role in rehabilitation from their home environment, TR reduces dependence on healthcare professionals for routine care, thereby strengthening self-efficacy and motivation to adhere to treatment [63]. Although we acknowledge that these considerations remain speculative, as the present study did not directly assess usability, adherence, and motivation, they are consistent with our previous work showing HomeCoRe’s usability [32] and that more than half of participants favored the home-based solution [31]. Taken together, these findings suggest that engagement with the TR software reflects intrinsic motivation and supports adherence. Finally, the lack of improvement in global cognition with the CoRe approach may indicate that HomeCoRe fosters greater patient engagement and, possibly, satisfaction.

Considering all the other cognitive domains, we found a significant improvement in working memory and logical-executive functions for both groups, in line with our previous experience with the CoRe system [24, 25]. Conversely, we found no significant change in episodic long-term memory and attention/processing speed in both groups. These results could be explained by the nature of the cognitive intervention administered, i.e., an adaptive process-based cognitive training (PCT), which aims at stimulating neuronal plasticity through the repetitive practice of training tasks [64], without explicit strategies. Previous studies provided evidence to support the efficacy of PCT in enhancing logical-executive functions [64, 65] and working memory [66], by decreasing the workload for metabolic and mental resources, represented at the functional level by decreased activity in the dorsolateral prefrontal cortex (DLPFC) [65, 66]. On the other hand, strategy-based cognitive training (SCT) has been shown to be particularly effective for the improvement of memory [67, 68] and attention/processing speed [69]. However, SCT has been shown to have scarce applicability in older adults with mild cognitive decline [70, 71].

Regarding behavioral and well-being outcomes, depressive symptoms and mental health status remained stable across time and groups. This stability may be attributed to the baseline characteristics of the sample, as both groups exhibited low levels of depressive symptoms and moderate to good perceived mental health at T0. It remains possible that subtle or delayed benefits on psychological well-being could emerge over time. Notably, a significant change in self-perceived physical health was observed only in the HomeCoRe group. However, significant baseline differences between the two groups limit the interpretability of this finding; therefore, follow-up assessments will be essential to interpret these preliminary results.

Finally, as for participant-centered outcomes, all participants reported a good perception of cognitive changes after both HomeCoRe and CoRe interventions. We acknowledge that participants’ awareness of their allocation may represent a potential source of bias; however, the absence of blinding was inherent to the study design, which was aimed at comparing two delivery modalities of the same intervention. Nonetheless, we believe subjective outcomes were unlikely to be influenced, as they referred to individual experiences of change within groups, without requiring cross-group comparisons. Moreover, objective measures supported these subjective reports, as we found a significant improvement in global WSs for both groups, in line with our previous experience with CoRe [24, 25], as well as with usability assessments of the HomeCoRe system [32]. These findings may stem from the fact that both CoRe and HomeCoRe were developed according to specific requirements provided by clinicians to address the specific needs and characteristics of older adults with cognitive decline, to facilitate the integration of TR services in clinical practice. Furthermore, it is necessary to address the larger effect size observed in the CoRe group compared to the HomeCoRe group, which may be attributed to the supervision of the therapist during the in-clinic intervention. This finding highlights the crucial role of the therapist in sustaining clinical practice, as their presence provides not only professional supervision but also motivational support during rehabilitation [72], thus enhancing the benefits. Importantly, therapists and caregivers provide different types of support, which constitutes an inherent distinction between the two delivery modalities. However, the main aim of the present study was not to demonstrate TR’s superiority, but rather to evaluate TR’s non-inferiority. One modality should not replace the other; instead, both solutions can be integrated into clinical practice, thereby offering the opportunity to choose the best-suited intervention.

In line with a previous systematic review and meta-analysis [5], we demonstrated that HomeCoRe’s benefits are non-inferior to its in-person version CoRe. This outcome supports the use of HomeCoRe in the clinical setting, allowing patients to choose the rehabilitation delivery modality that best aligns with their individual needs and preferences.

Beyond its clinical effectiveness, HomeCoRe addresses several barriers that still limit TR integration into clinical practice. The simple, intuitive, and user-friendly interface of HomeCoRe appears to be well-suited for older adults, overcoming the limits related to this population’s characteristics, such as poor technological skills and their reluctance to adopt digital tools [8, 10], and appearing useful and enjoyable to its users [32]. Furthermore, HomeCoRe facilitated the inclusion of a broader and less digitally literate sample by providing participants with pre-configured laptops and supporting both online and offline functionalities, thereby removing the requirement for personal device ownership or home internet access demanded by previous studies [13–17]. Moreover, HomeCoRe was also designed to minimize the need for caregivers’ support during rehabilitation sessions, promoting a more sustainable and supportive therapeutic environment.

Importantly, this study represents the next step in the development of the CoRe/HomeCoRe systems. Building on a series of previous trials that established the usability and efficacy of CoRe [23–26], the present trial extends this research by assessing the non-inferiority of HomeCoRe compared to its validated in-person counterpart. This strengthens the overall evidence supporting the CoRe/HomeCoRe software and contributes uniquely to the field by demonstrating that a previously validated cognitive intervention can be effectively delivered through remote rehabilitation.

Finally, to the best of our knowledge, this is the first RCT on cognitive TR that directly compared different delivery modalities (i.e., TR versus in-person) for the same cognitive intervention, providing novel evidence on the clinical effectiveness of TR and supporting its integration into routine care.

This study has some limitations. First, we reported data on cognitive, physical, and mental health outcomes at T1 only; therefore, follow-up assessments at T2 and T3 are needed to verify the long-term efficacy of HomeCoRe in older adults with cognitive decline. This data has already been collected and will be presented in future work. Moreover, our analyses focused exclusively on behavioral outcomes; thus, future studies should integrate structural and functional measures to provide a more comprehensive understanding of the effects of TR at the neural level. In fact, additional work is ongoing to investigate the neural correlates of HomeCoRe’s effects, as described in the protocol by Caminiti et al. [73]. Another limitation is the absence of direct measures of usability, adherence and motivation, which were beyond the primary aims of this study. Nonetheless, previous work showed that HomeCoRe is accessible and user-friendly [32], and that over half of participants preferred the home-based modality [31], suggesting good engagement and intrinsic motivation toward TR. Finally, we acknowledge that excluding participants without a caregiver limits the generalizability of our findings. However, this restriction was necessary to guarantee comparable conditions for participant enrollment and randomization across the two groups. Moreover, the requirement of a caregiver represents an intrinsic limitation of TR, reinforcing its role as a complementary option suitable for patients meeting specific eligibility criteria. The involvement of a caregiver would remain essential in real-world clinical implementation, providing critical support during the rehabilitation plan.

This study offers timely and concrete evidence on how the delivery modality can shape cognitive rehabilitation outcomes. To our knowledge, it is among the first RCTs to compare the same intervention provided either in person or remotely. Demonstrating the non-inferiority of HomeCoRe supports a more flexible, patient-centered approach to cognitive care, one that is both clinically effective and feasible in real-world settings. Based on these findings, we are currently developing a mobile app version of HomeCoRe, with the goal of expanding access to cognitive training for a broader and more diverse population. Future research will be crucial to evaluate the usability and effectiveness of this app-based solution and to support its integration into everyday clinical practice.

## Data Availability

The datasets presented in this study can be found in online repositories. The names of the repository/repositories and accession number(s) can be found below: 10.5281/zenodo.15389025.
